# Use of a Recombinant Vaccinia Virus Expressing Interferon Gamma for Post-Exposure Protection against Vaccinia and Ectromelia Viruses

**DOI:** 10.1371/journal.pone.0077879

**Published:** 2013-10-17

**Authors:** Susan A. Holechek, Karen L. Denzler, Michael C. Heck, Jill Schriewer, R. Mark Buller, Fatema A. Legrand, Paulo H. Verardi, Leslie A. Jones, Tilahun Yilma, Bertram L. Jacobs

**Affiliations:** 1 Center for Infectious Diseases and Vaccinology, The Biodesign Institute, Arizona State University, Tempe, Arizona, United States of America; 2 Department of Molecular Microbiology and Immunology, Saint Louis University Health Sciences Center, St. Louis, Missouri, United States of America; 3 International Laboratory of Molecular Biology for Tropical Disease Agents, School of Veterinary Medicine, Department of Medical Microbiology and Immunology, School of Medicine, University of California, Davis, California, United States of America; 4 Department of Pathobiology and Veterinary Science, University of Connecticut, Storrs, Connecticut, United States of America; Indian Institute of Science, India

## Abstract

Post-exposure vaccination with vaccinia virus (VACV) has been suggested to be effective in minimizing death if administered within four days of smallpox exposure. While there is anecdotal evidence for efficacy of post-exposure vaccination this has not been definitively studied in humans. In this study, we analyzed post-exposure prophylaxis using several attenuated recombinant VACV in a mouse model. A recombinant VACV expressing murine interferon gamma (IFN-γ) was most effective for post-exposure protection of mice infected with VACV and ectromelia virus (ECTV). Untreated animals infected with VACV exhibited severe weight loss and morbidity leading to 100% mortality by 8 to 10 days post-infection. Animals treated one day post-infection had milder symptoms, decreased weight loss and morbidity, and 100% survival. Treatment on days 2 or 3 post-infection resulted in 40% and 20% survival, respectively. Similar results were seen in ECTV-infected mice. Despite the differences in survival rates in the VACV model, the viral load was similar in both treated and untreated mice while treated mice displayed a high level of IFN-γ in the serum. These results suggest that protection provided by IFN-γ expressed by VACV may be mediated by its immunoregulatory activities rather than its antiviral effects. These results highlight the importance of IFN-γ as a modulator of the immune response for post-exposure prophylaxis and could be used potentially as another post-exposure prophylaxis tool to prevent morbidity following infection with smallpox and other orthopoxviruses.

## Introduction

Vaccinia virus (VACV) is a member of the genus Orthopoxvirus of the family Poxviridae. Other members of this family include variola virus (VARV), the causative agent of smallpox; monkeypox virus (MPXV), ectromelia virus (ECTV), camelpox virus (CMLV) and cowpox virus (CPXV) [1][2]. After an intensive vaccination strategy using VACV, which was initiated in 1967, smallpox was declared eradicated from the world in 1980 and routine smallpox vaccination was discontinued in the USA in 1972 [3][4]. This leaves the majority of the current world population susceptible to severe disease from certain orthopoxvirus infections. After September 11, 2001, there was heightened concern for the use of VARV or MPXV as a bioterrorism agent and the low, but real, risk of an accidental VARV release [5][6][7]. For these reasons it was important to develop a post-exposure prophylaxis strategy. 

There are diverse antiviral agents that have been considered as alternative strategies for post-exposure treatment of smallpox. One of the antiviral drugs that has been shown to be effective against different poxviruses is (S)-1-(3-hydroxy-2-phosphonylmethoxypropyl)-cytosine also known as HPMPC, Vistide, and more commonly as cidofovir [8][9]. While cidofovir has been shown to protect monkeypox-infected macaques when administered 24 hours post-infection (hpi), its potential is limited by nephrotoxicity and the lack of oral availability [9]. Likewise, vaccine immune globulin (VIG), has also been suggested to be used as prophylaxis in patients for whom smallpox vaccine is contraindicated [10]. 

Antivirals ST-246 and CMX001 have shown promise as antipoxviral drugs and are in phase I and II clinical trials [11][12][13][14][15][16]. ST-246 protects ground squirrels against monkeypox challenge when administered up to 3 days post-exposure [17]. Treatment with VIGIV (Vaccinia Immune Globulin Intravenous) and cidofovir along with ST-246 (multiple doses) was successful in the treatment of a 28-month-old child with severe eczema vaccinatum [18][19]. Similarly, a combination therapy using VIGIV, ST-246, Imiquimod and CMX001, a lipid conjugate of cidofovir, were used to treat progressive vaccinia in a military vaccinee [20]. Moreover, VIGIV and ST-246 have recently been used to treat human vaccinia virus infection in a 35 year-old woman after contact with raccoon rabies bait [21]. Although it is difficult to assess the contribution of each agent because of the close timing of administration, these cases are an example of the utility of new treatments.

Post-exposure vaccination with VACV has also been suggested to be an effective treatment if administered within four days of smallpox exposure [22]. While there is anecdotal evidence for efficacy of post-exposure vaccination this has not been definitively studied in humans. Therefore, various animal models have been used to study the efficacy of post-exposure treatment with various VACV strains following poxvirus infection. While infection of humans with smallpox has a 14 day period from initial infection to the prodrome phase, the various animal models show pathology via weight loss within 3-4 days following infection. Therefore the time during which post-exposure prophylaxis can occur is abbreviated in these models. 

A recent model of post-exposure immunization with modified VACV Ankara (MVA) and the VACV-Lister strain proved to be effective in an ECTV infection model when post-exposure vaccination was performed at 1, 2 and 3 days post-infection (dpi). Protection was shown to be dependant on the vaccine dose as well as the day of vaccination [23]. A second model employs the Western Reserve (WR) strain of VACV as a challenge virus in mice. Therapeutic vaccination with MVA was minimally protective only at 1 dpi while the Elstree (Lister) strain failed to provide any detectable protection [24]. 

Because cytokines are highly regulated during VACV infection, they have been thoroughly studied in this context [25][26][27][28]. While IL-2, TNF-α and IFN-γ decrease virulence during a VACV infection, others such as IL-4 have been shown to increase virulence and pathogenicity [25][26][27][28]. Another cytokine, IL-18, which can induce endogenous synthesis of IFN-γ, has been shown to promote clearance of VACV infection when expressed together with IL-12, indicating the synergistic action of both cytokines[29]. 

The present work employed recombinant VACV for post-exposure prophylaxis in an animal model. We evaluated several recombinant VACVs as therapeutic vaccines including 1) viruses containing mutations within the VACV E3L gene, an IFN resistance gene [30][31] and 2) viruses expressing murine IFN-γ (muIFN-γ) [32] or murine IL-18 (muIL-18) from the VACV B13R locus. In this study we demonstrate that post-exposure prophylaxis with a recombinant VACV expressing IFN-γ is 100% effective in protecting mice infected with 100 LD_50s_ of wt VACV as well as 83.3% effective in protecting mice infected with 55 LD_50s_ of ECTV when administered 1 dpi. 

## Materials and Methods

### Ethics statement

Animal experiments performed at Arizona State University were approved and carried out under the IACUC protocol number 08-970R. Animal husbandry and experimental procedures performed at Saint Louis University were in accordance with Public Health Service policy, and approved by Animal Care and Use Committee. The IACUC protocol number 1339 was followed for these studies.

### Viruses and cells

#### VACV

The WR strain of VACV was used as the parental virus for these studies. The E3L VACV mutants were generated by standard methods routinely employed in our laboratory. Construction of VACV mutants expressing E3LΔ7C and E3LΔ26C has previously been described [33]. VACVΔE3L::ATVeIF2α has a replacement of the E3L gene with the eukaryotic initiation factor 2 alpha (eIF2α) homologue from the *Ambystoma tigrinum* virus, genus *Ranavirus*, family Iridoviridae [34]. The recombinant VACV expressing IFN-γ (v50ΔB13RMγ) virus was constructed as previously described [32]. This virus expresses the vesicular stomatitis virus glycoprotein (VSV-G) at the TK locus and *lacZ*, gpt, and muIFN-γ at the B13R site, thus both VACV genes are inactivated. The recombinant VACV expressing IL-18 (v50ΔB13RMIL‑18) is essentially the same as v50ΔB13RMγ, but expresses the muIL-18 gene instead of muIFN-γ. The muIL‑18 gene was cloned from mouse spleen cDNA (unpublished data). Construction of the parental virus (v50ΔB13R) has already been described [35]. Viruses were amplified in Baby Hamster Kidney 21 (BHK) cells and partially purified by pelleting through a 36% sucrose pad, as previously described [36][37]. BHK and Rabbit Kidney 13 (RK) cells were cultured in minimal essential medium (MEM, Gibco, BRL) containing 5% fetal bovine serum (FBS), 50 μg/ml of gentamycin, 0.1 mM nonessential amino acid solution (Gibco, BRL), and vitamin supplements. Cells were incubated at 37°C with 5% CO_2_. 

#### ECTV

African green monkey kidney epithelial cells (BSC-1, ATCC CCL-26) were grown in Eagle’s MEM containing 10% fetal clone III serum (Hyclone, Logan, UT), 2mM L-glutamine (GIBCO, Grand Island, NY), 100 U/ml penicillin (GIBCO, Grand Island, NY), and 100 μg/ml streptomycin (GIBCO, Grand Island, NY). A plaque-purified isolate of the MOS strain of ECTV (ATCC VR-1374) designated MOS-3-P2 was propagated in BSC-1 cells [38]. Virus was partially purified through a sucrose cushion [39], and infectivity was estimated, as described previously [40]. Briefly, virus suspensions were serially diluted in phosphate buffered saline (PBS) + 1% FBS, absorbed to monolayers for 1 h at 37°C, and overlaid with a suspension of 1% carboxymethyl cellulose in DMEM+ 5% fetal clone III serum. After 4 days at 37°C, plaques were visualized by staining with a 0.3% crystal violet/10% formalin solution.

### Mice and *in vivo* infections

#### VACV infections

Four-week old female C57BL/6 mice were obtained from Charles River and maintained at the Arizona State University Department of Animal Care and Technologies. Each cage contained a maximum of 5 mice and a separate cage was used for each experimental condition. Treatment groups consisted of 5 to 15 mice. An anesthetic cocktail containing xylazine (7.5 mg/ml), acepromazine maleate (2.5 mg/ml), and ketamine (37.5 mg/ml) was prepared. Approximately 1 μl of cocktail was injected intramuscularly per gram of body weight [31]. Following anesthesia, one naris was infected with a 5 μl dose of 10^6^ pfu (~100 LD_50s_) of wt VACV. Mice were treated 1, 2 or 3 dpi with 10^2^ - 10^7^ pfu of treatment virus administered intranasally (IN) into the same or opposite naris as the challenge virus in a 5 ml volume. Scarification infection of mice was done as previously described [41]. IM injections were performed by injecting 5 ml of virus suspension into the left leg using a 26-gauge needle. Disease symptoms and animals’ health were monitored every other day for the length of the experiment. 

#### ECTV infections

Eight to ten-week-old male or female C57BL/6 mice were obtained from the National Cancer Institute, (Frederick, MD), housed in filtertop microisolator cages and fed commercial mouse chow and water, *ad libitum*. The mice were housed in an animal biosafety level 3 containment area. One day prior to challenge with ECTV, individual mice were weighed and assigned to treatment groups of 10 to 20 mice. On the day of challenge, mice were anesthetized with 0.1 ml/10 g body weight of ketamine HCl (9 mg/ml) and xylazine (1 mg/ml) by intraperitoneal injections. Anesthetized mice were laid on their dorsal side with their bodies angled so that the anterior end was raised 45° from the surface. A plastic mouse holder was used to ensure conformity. ECTV was diluted in PBS to the required dose (5 x 10^3^ pfu) and slowly loaded into each naris (5 μl/naris). Mice were subsequently left in situ for 2–3 min before being returned to their cages. One day following challenge, groups of mice were treated by the foot pad (FP) route with 3 x 10^7^ pfu of v50ΔB13RMγ or v50ΔB13R. Mice were weighed every 2 days and observed for morbidity and mortality. 

### Weight loss

Weight loss was determined by weighing each mouse on alternate days. The percent weight gain or loss was determined and animals that lost more than 30% of their original body weight were euthanized by an IP injection of anesthesia or by CO_2_ inhalation and considered dead from the challenge. A relative sickness index that takes into consideration typical symptoms of a wt VACV infection was created. Each animal from each group was assigned an arbitrary score (1-4) based on the severity of the following symptoms: ruffled fur, lack of activity, breathing difficulty, eye infection, hunching and weight loss. This index also takes into consideration the number of animals euthanized within each group (each death accounts for a value of 0.8 in the death/life score, if all animals in the group die, the line stops). 

### Tissue distribution

Animals were infected IN with 10^6^ pfu of wt VACV (Day 0) and treated with 10^7^ pfu of v50ΔB13RMγ. On alternate days beginning with 2 dpi, three animals were euthanized and then immediately dissected. The organs removed (nasal cavity, brain, lungs, heart, liver, spleen, kidney and ovaries) were immediately frozen in liquid nitrogen and then stored at -80°C. A 10% homogenate was prepared for each organ by adding 1 mM Tris pH 8.8 with gentamycin. All tissues with the exception of the nasal cavity were then homogenized using a PCR tissue homogenizing kit (Fisher). The nasal cavity was homogenized using a Mixer Miller 301 device (Retsch). All homogenates were subjected to three rounds of freezing (−80°C), slow thawing for 30 min on ice and then quick thawing (37°C). After three rounds of freezing-thawing, samples were subjected to a 7 minute spin using a tabletop centrifuge at 700 x *g* at 4°C to remove all cell debris. After centrifugation, supernatants were retained and dilutions were performed for plaque assays on RK13 cells. 

Viral loads for tissue distribution were conducted in triplicate by infecting monolayers of RK cells. Forty-eight hours post-infection, two replicates were stained with crystal violet to determine the viral load within the tissue (pfu per g of tissue). Differentiation of wt VACV and the treatment v50ΔB13RMγ virus was done by overlaying the third replica with 1% agarose, MEM 5% FBS and 400 μg/ml X-Gal (5-bromo-4-chloro-3-indolyl-β-D-galactopyranoside). The v50ΔB13RMγ could be identified due to the fact that this virus expresses *lacZ* from the B13R locus. 

### ELISA

Animals were infected on day 0, treated on day 1, and 24 hours later blood was collected from the superficial temporal vein into BD serum separator tubes. Serum was collected after centrifugation following manufacturer’s recommendations and stored at -80°C. An ELISA to detect murine IFN-γ was performed using a kit from Thermo Fisher. Briefly, plates precoated with IFN-γ antibody were incubated with serum samples diluted 1:2 in standard diluent, washed, and incubated with a second biotin-conjugated IFN-γ antibody. After washing, streptavidin-HRP was added, and detection was performed using TMB substrate solution. The reaction was stopped with dilute sulfuric acid and a plate reader was used to measure the absorbance at 450nm. 

### Immunohistochemistry

Tissues were perfused with phosphate-buffered saline (PBS) via cardiac injection followed by fixation with 10% formalin. After the soft tissue was removed the skull was decalcified using ethylene glycol tetraacetic acid (EGTA). The skull was embedded in paraffin and sliced at a 3 μm thickness using a microtome starting at the tip of the nasal cavity and moving in an anterior direction. Deparaffinized tissue sections of the nasal cavity were incubated with 2% H_2_O_2_ in methanol for 10 minutes to quench endogenous peroxidases and then rinsed in PBS. Detection of VACV antigen was done using rabbit anti-VACV and the VECTASTAIN Elite ABC kit (Rabbit IgG) from VECTOR Laboratories. Color development was performed using the 3,3'-diaminobenzidine (DAB) Substrate Kit from VECTOR Laboratories following the manufacturer's protocol. 

### Data analysis

Survival analysis was done using Kaplan-Meier curves and the log-rank test and Mann-Whitney analyses were performed using the statistical software program GraphPad Prism (version 5.0c for Macintosh).

## Results

### v50ΔB13RMγ causes weight recovery and protects mice from death

The WR strain of VACV was originally derived from serial passages in mouse brain and thus is a neurotropic, highly virulent strain of VACV [42]. Several VACV WR mutants of E3L (an IFN antagonist protein) as well as VACV WR recombinants expressing either muIL-18 or muIFN-γ were tested for their ability to confer protective post-exposure prophylaxis in mice upon challenge with a lethal dose of VACV WR. The VACV E3L mutants chosen for this study have previously been shown to have reduced pathogenesis and neurovirulence phenotypes in mice [31]. Similarly, IL-18 was selected due to its pro-inflammatory properties, ability to induce synthesis of IFN-γ, activation of NK cells and its main role in the T-lymphocyte helper type 1 response [43]. IFN-γ was chosen due to its multiple immune stimulatory effects on macrophage activities, NK cell cytolysis, and T and B cell responses [26][44][45][46][47].

**Figure 1:**
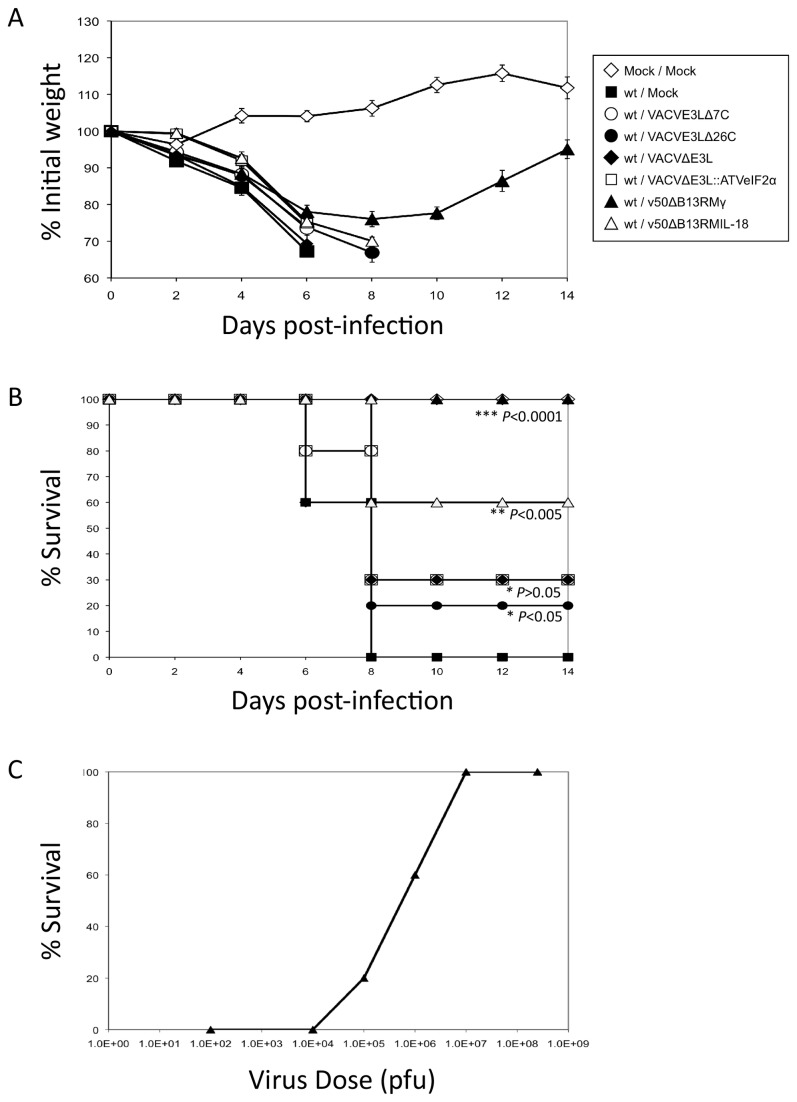
Post-exposure protection of animals after intranasal infection with a lethal dose of wt VACV and subsequent treatment with different VACV mutants. (A) Weight loss. Groups of 5 to 10 four-week-old C57BL/6 mice were infected intranasally with 10^6^ pfu (~100 LD_50s_) of wt VACV and treated 1 dpi with 10^7^ pfu of VACVE3LΔ7C (○), VACVE3LΔ26C (●), VACVΔE3L (♦), VACVΔE3L::ATVeIF2α (□), v50ΔB13RMγ (▲) and v50ΔB13RMIL-18 (∆). There were two groups of control animals, one group was infected with wt VACV alone and was mock-treated (■), the second group of animals was mock-infected and mock-treated (◊). Each mouse was weighed at the indicated times. Average percentage of initial weight of the animals infected with each virus is plotted versus time (days post-infection). Lines end at the death of one animal. Error bars indicate the standard error of the mean. (B) Survival curve. The data represent a pool of two independent experiments using group sizes of 5 mice. (C) v50ΔB13RMγ dose response. Groups of 10 four-week-old C57BL/6 mice were infected intranasally with 10^6^ pfu of wt VACV (~100 LD_50s_) and treated one day post-infection with doses of 10^2^, 10^3^, 10^4^, 10^5^, 10^6^, 10^7^ and 5 x 10^8^ pfu of v50ΔB13RMγ (▲).

IN infections of C57BL/6 mice were performed with ~100 LD_50s_ wt VACV as described in the materials and methods section. Animals were then treated 1 dpi with 10^7^ pfu of the mutant or recombinant viruses. As shown in Figure 1A, all animals lost weight to day 6 post-infection, but only mice treated with v50ΔB13RMγ recovered. Figure 1B shows the survival rate for each of the mutants tested. Treatment with v50ΔB13RMIL-18 resulted in 60% survival (P<0.005, log-rank test) while 30% survival was observed when animals were treated with either VACVΔE3L, VACVE3LΔ7C or VACVE3L::ATVeIF2α (P>0.05, log-rank test) and 20% survival when treated with VACVE3LΔ26C (P<0.05, log-rank test). Only treatment with v50ΔB13RMγ was able to confer 100% survival (P<0.0001, log-rank test). Partial protection was seen with doses as low as 10^5^ pfu, while full protection required treatment with 10^7^ pfu (Figure 1C). 

The parental virus of v50ΔB13RMγ, v50ΔB13R, was also evaluated in order to assess if the protection from mortality was a result of the expression of IFN-γ or whether it was a result of the parental virus containing a deletion of the serpin gene B13R. This gene is nonessential for VACV replication, however deletion from the virus results in reduced replication and pathogenesis in mice [35]. Treatment of mice infected with wt VACV as described above with the parental virus (v50ΔB13R) was analyzed. This virus did not provide protection against infection with VACV with only 17% surviving (data not shown, P>0.5, log-rank test), suggesting that expression of IFN-γ and not the deletion of B13R, is responsible for the survival of the mice.

### Post-Exposure Prophylaxis with v50ΔB13RMγ Is More Effective When Administered One Day Post-Infection

**Figure 2:**
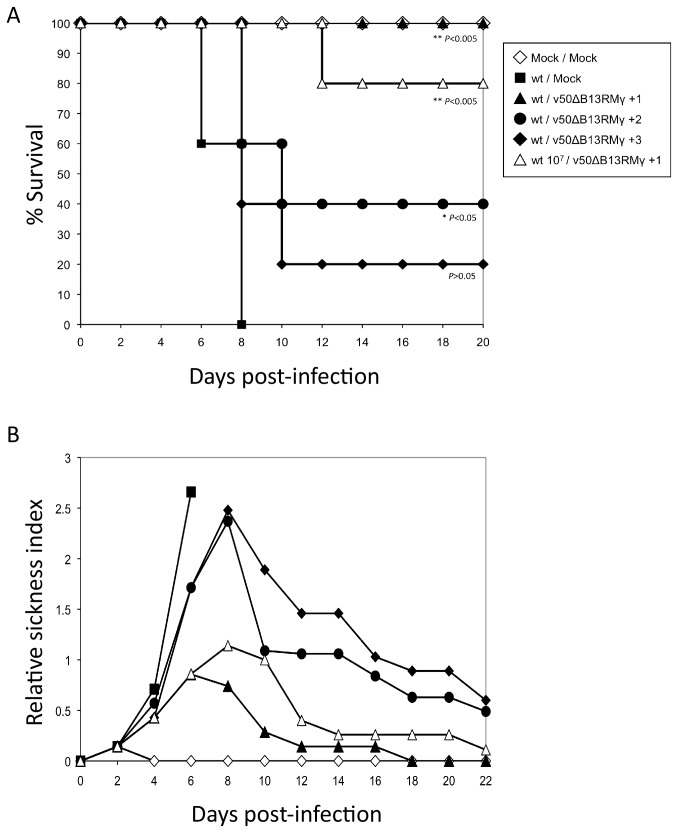
Protection of wt VACV infected animals by post-exposure vaccination with v50ΔB13RMγ at one, two and three days post-infection. Groups of 5 to 10 C57BL/6 mice were infected intranasally with 10^6^ pfu (~100 LD_50s_) of wt VACV. Animals were treated with 10^7^ pfu of v50ΔB13RMγ at one (▲), two (●) or three (♦) days post-infection. One group was infected with 10^7^ pfu (~1000 LD_50s_) of wt VACV and treated with 10^7^ pfu of v50ΔB13RMγ at one day post-infection (∆). Controls animals were infected with wt VACV and then mock-treated (■), or animals were mock-infected and mock-treated (◊). (A) Comparison of survival curves was done using the log-rank test. (B) Recovery of wt VACV infected animals by post-exposure vaccination with v50ΔB13RMγ at one, two and three days post-infection. The graph indicates the relative sickness of each group of animals during the course of the infection. Lines ending prematurely indicate death of all the animals from the group. A value of 0 indicates that all the animals from that group were healthy.

In order to determine the temporal requirements of protection, animals were infected with 10^6^ pfu (~100 LD_50s_) of wt VACV and then treated with 10^7^ pfu of v50ΔB13RMγ at 1, 2, or 3 dpi. Animals showed 100% survival when treated 1 dpi, while all mock-treated animals died by day 8 (P<0.005, log-rank test). Protection was reduced to 40% (P<0.05, log-rank test) when treatment virus was administered at 2 dpi, and to 20% (P>0.05, log-rank test) when animals were treated at 3 dpi (Figure 2A). Additionally, when animals were initially infected with ten-fold more wt VACV (10^7^ pfu or 1000 LD_50s_) followed by treatment 1 dpi with v50ΔB13RMγ a slight decrease to 80% survival was observed (P<0.005, log-rank test). 

*A relative* sickness index with values from 0 to 4 was created as indicated in the materials and methods section. Animals given 1 x 10^6^ pfu of wt VACV without therapeutic vaccination reached a relative sickness index of 2.7 by 6 dpi and succumbed to the infection by 8 dpi. However, mice that were treated with 10^7^ pfu v50ΔB13RMγ at 1 dpi reached a maximum relative sickness of 0.9 by 6 dpi. By day 18, these mice attained weights that were comparable to the mock-infected mice (data not shown) and had a relative sickness index of 0. Interestingly, animals that were treated either 2 or 3 dpi showed similar morbidity and had a sickness index similar to that of the untreated animals at 8 dpi, (2.5 versus 2.7). However, when animals were infected with 10^7^ pfu of wt VACV and treated 1 dpi, they reached a maximum score of 1.1 by 8 dpi and the animals that survived showed a slightly delayed recovery as compared to mice infected with 10^6^ pfu wt VACV and treated 1 dpi (Figure 2B).

### The intranasal route of administration of post-exposure prophylaxis with v50ΔB13RMγ is most effective

**Figure 3:**
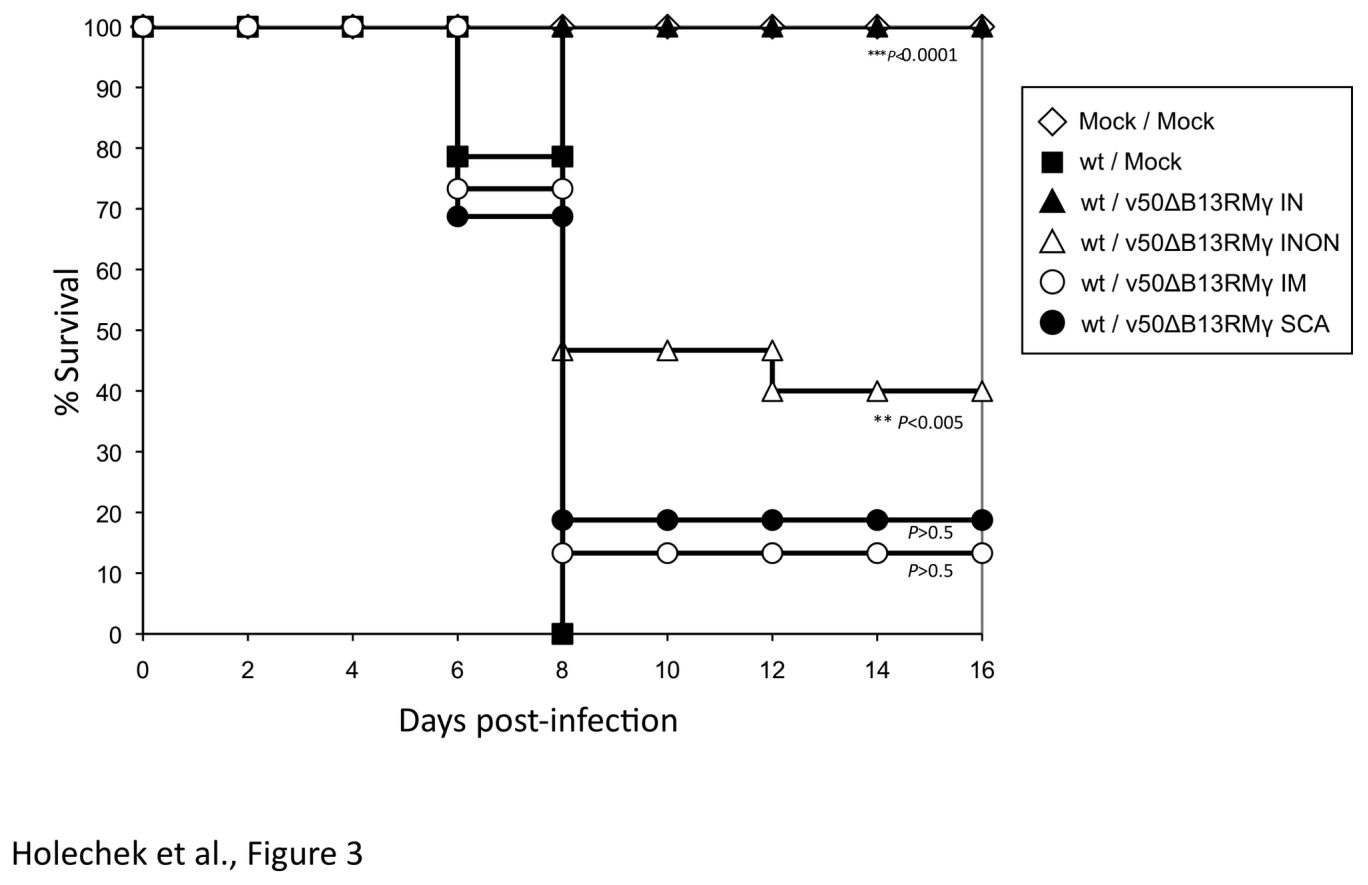
Post-exposure protection of wt VACV infected animals treated with v50ΔB13RMγ using different routes of treatment. Groups of 12 to 15 C57BL/6 mice were infected intranasally with 10^6^ pfu of wt VACV. Animals were treated one day post-infection with 10^7^ pfu of v50ΔB13RMγ intranasally, IN (▲), intranasally using the other nostril, INON (∆), intramuscularly, IM (○) or via scarification, SCA (**●**). One group of animals was infected with wt VACV and then mock-treated (■), another group was mock-infected and mock-treated (◊). Comparison of survival curves was done using the log-rank test.

In the previous experiments, all viruses were administered into the same naris of the animal. Since the current smallpox vaccine is given by scarification and several vaccines are administered by intramuscular (IM) injection, animals were challenged IN as described above and treated with v50ΔB13RMγ into the same naris (IN), opposite naris (INON), by scarification (SCA) or by IM injection. The results show that 100% protection occurred only when the treatment was given into the same naris as the challenge virus (P<0.0001, log-rank test) (Figure 3). On the other hand, mice that remained untreated had marked weight loss, morbidity and died 8 dpi. INON treatment resulted in 40% survival (P<0.005, log-rank test), and IM or SCA treatment resulted in 20% and 15% survival, respectively (P>0.5, log-rank test) (Figure 3). As in previous experiments, a relative sickness index was generated. Animals that were treated within the same naris had the lowest relative sickness index, 0.6 at 8 dpi, while treatment in the opposite naris had an index of 1.74 and treatment via SCA or IM injection had the highest sickness index of 2.0 by 8 dpi, similar to untreated animals (data not shown). 

### Viral spread of wt VACV was similar in untreated and treated animals

**Figure 4:**
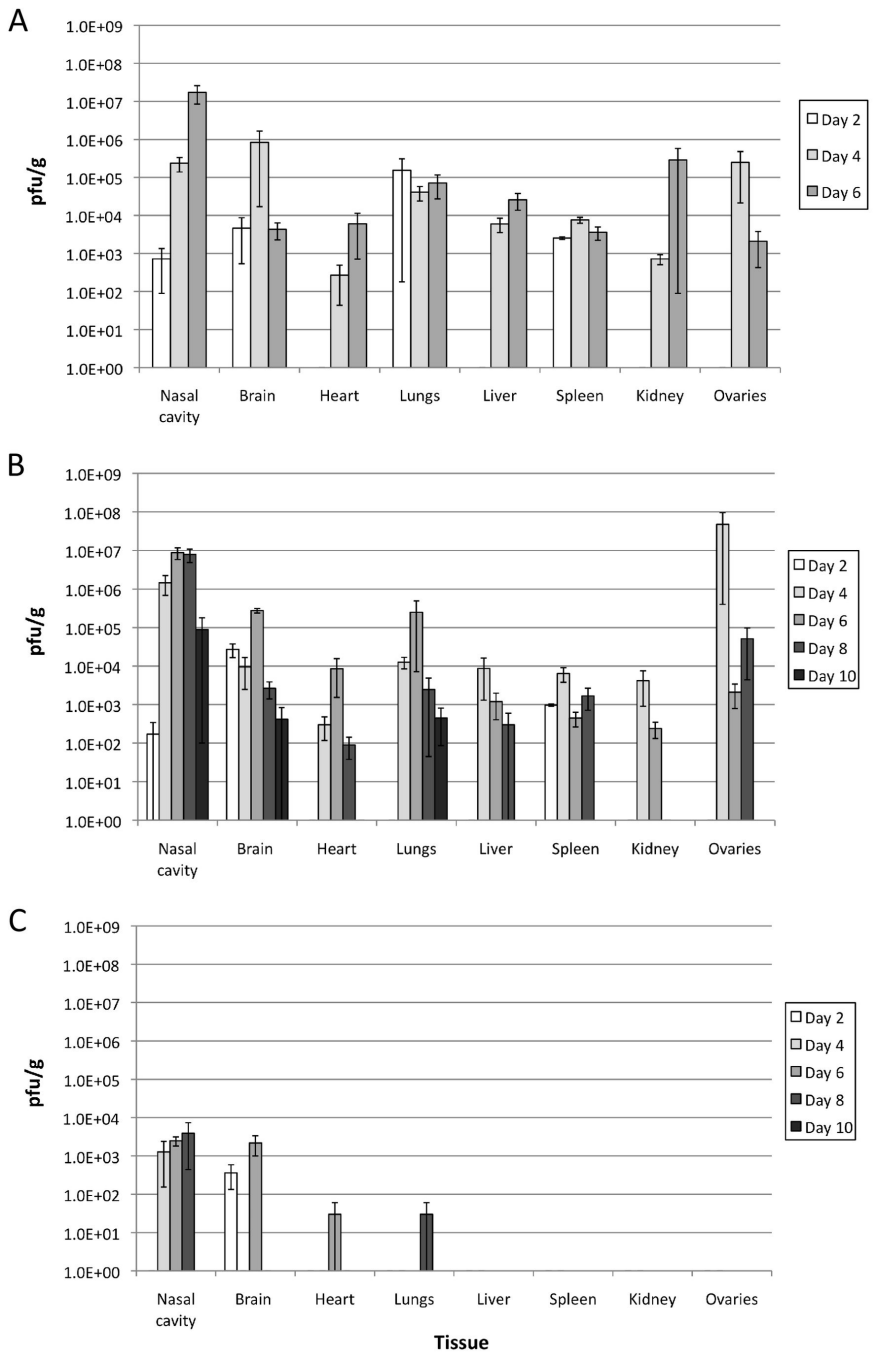
Viral spread of wt VACV in infected animals. Groups of 3 C57BL/6 mice were infected intranasally with 10^6^ pfu of wt VACV. Animals remained untreated (A) or were treated one day post-infection with 10^7^ pfu of v50ΔB13RMγ (B). Replication of v50ΔB13RMγ in the tissues from the treated group was examined by X-gal staining (C). Error bars indicate the standard error of the mean. Data represent a pool of two independent experiments using 3 mice per group.

IFN-γ is both an effective antiviral as well as a modulator of the immune response by leading to the activation of macrophages and neutrophils and to increased expression of MHC class I and II proteins [26][44] which could limit the spread of VACV and lead to protection. To determine the kinetics of replication and viral spread, various mouse tissues (nasal cavity, brain, lungs, heart, liver, spleen, kidney and ovaries) were examined. At 2 dpi wt VACV was observed in the nasal cavity, brain, lungs and spleen of mock-treated animals (Figure 4A). Animals that were treated with v50ΔB13RMγ showed similar viral loads in the nasal cavity, brain and spleen as in untreated animals, but no virus was detected in the lungs at 2 dpi (Figure 4B). By 4 dpi wt VACV was detected in all of the organs examined with similar titers in most tissues in both untreated and treated animals (Figures 4A and 4B). However, at 4 dpi titers in the brain of treated animals were two logs lower than titers in the brain of untreated mice while titers in the ovaries of treated mice were two logs higher than in untreated mice. At 6 dpi, titers in the ovaries of treated and untreated mice were similar. By 6 dpi, viral loads in treated animals peaked within the nasal cavity, brain, heart, and lungs and reached titers close to those observed in untreated animals at 4 dpi. By 10 dpi VACV could only be detected in the nasal cavity, brain, and lungs indicating that the treated animals were effectively clearing the viral load (Figure 4B). X-gal staining was done in order to differentiate v50ΔB13RMγ from wild-type virus. Examination of the different tissues revealed a low level of replication of this virus in the nasal cavity (4-8 dpi) and brain (2 and 6 dpi), as well as detection in the heart and lungs of animals, one at 6 dpi and one at 8 dpi respectively (Figure 4C).

### Animals treated with v50ΔB13RMγ show increased levels of IFN-γ in the serum

**Figure 5:**
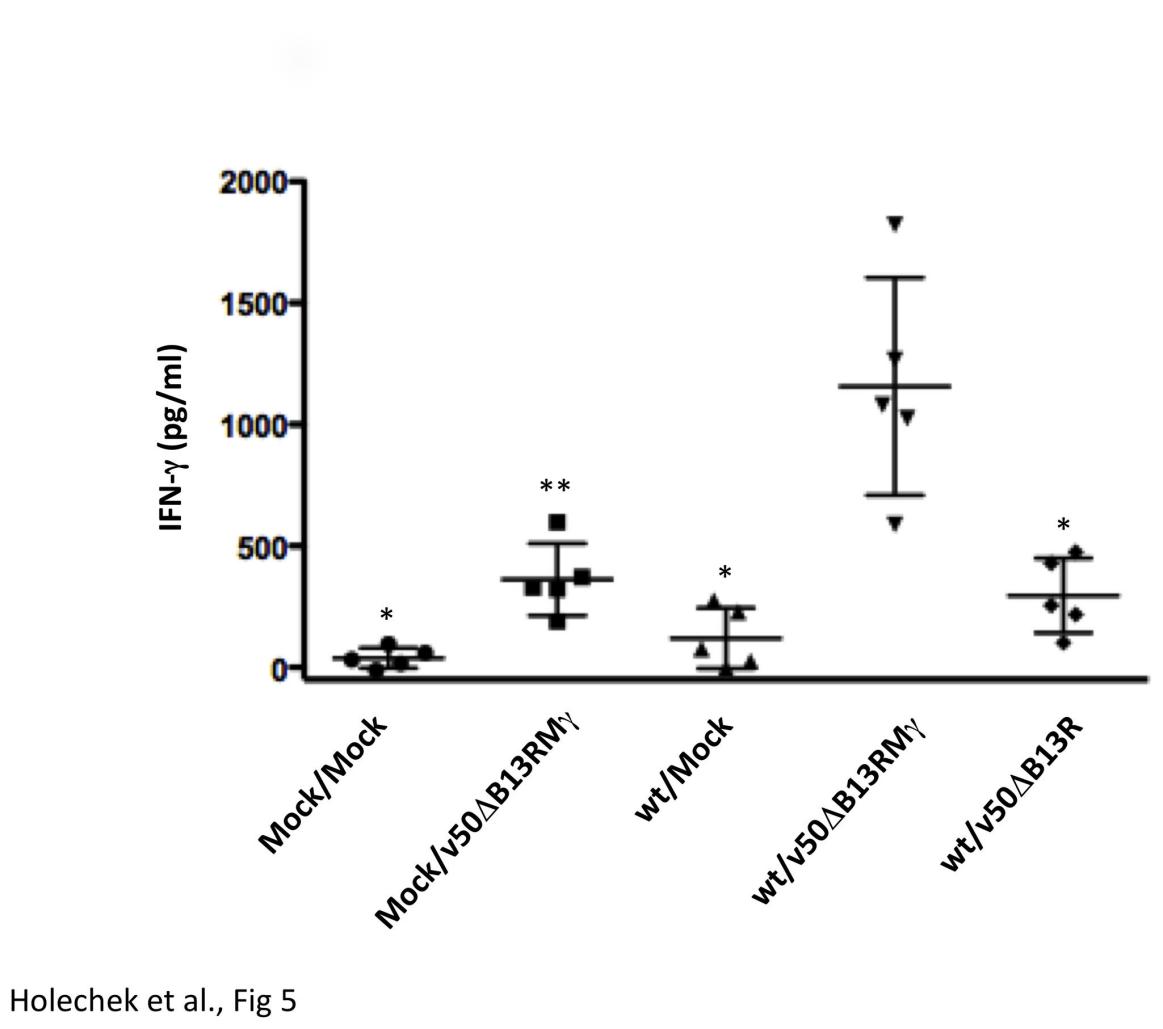
Measurement of IFN-γ in the serum following treatment. Groups of 5 C57BL/6 mice were mock-infected or infected IN with 10^6^ pfu (~100 LD_50s_) of wt VACV. One day post infection mice were either mock-treated or treated with 10^7^ pfu of v50ΔB13R or v50ΔB13RMγ. After 24 hours, serum was harvested and an ELISA was performed to measure IFN-γ. The horizontal line indicates the average and error bars indicate the standard error of the mean. Comparisons were done using the Mann-Whitney test. One asterisk (*) represents p<0.008 in comparison to the wt/v50ΔB13RMγ group. Two asterisks (**) represents p<0.016 in comparison to the wt/v50ΔB13RMγ group.

Serum was collected 24 hours following mock treatment or treatment with v50ΔB13R or v50ΔB13RMγ, and an ELISA was performed to measure IFN-γ. Mock-infected, mock-treated animals had the lowest levels of IFN-γ at 38 pg/ml, whereas treating these mice with v50ΔB13RMγ resulted in an increase to 362 pg/ml IFN-γ (Figure 5). Animals that were wt-infected, mock-treated showed slightly elevated IFN-γ at 120 pg/ml. When wt-infected animals were treated with the parental virus, v50ΔB13R, 296 pg/ml IFN-γ was observed, whereas animals that were treated with v50ΔB13RMγ showed the highest levels of IFN-γ with an average of 1156 pg/ml (Figure 5). The wt-infected, v50ΔB13RMγ-treated animals showed statistically significant differences in levels of IFN-γ in comparison to all the other treatment groups. 

### Animals treated with v50ΔB13RMγ show decreased tissue necrosis, edema, and epithelial sloughing into the nasal cavity

**Figure 6:**
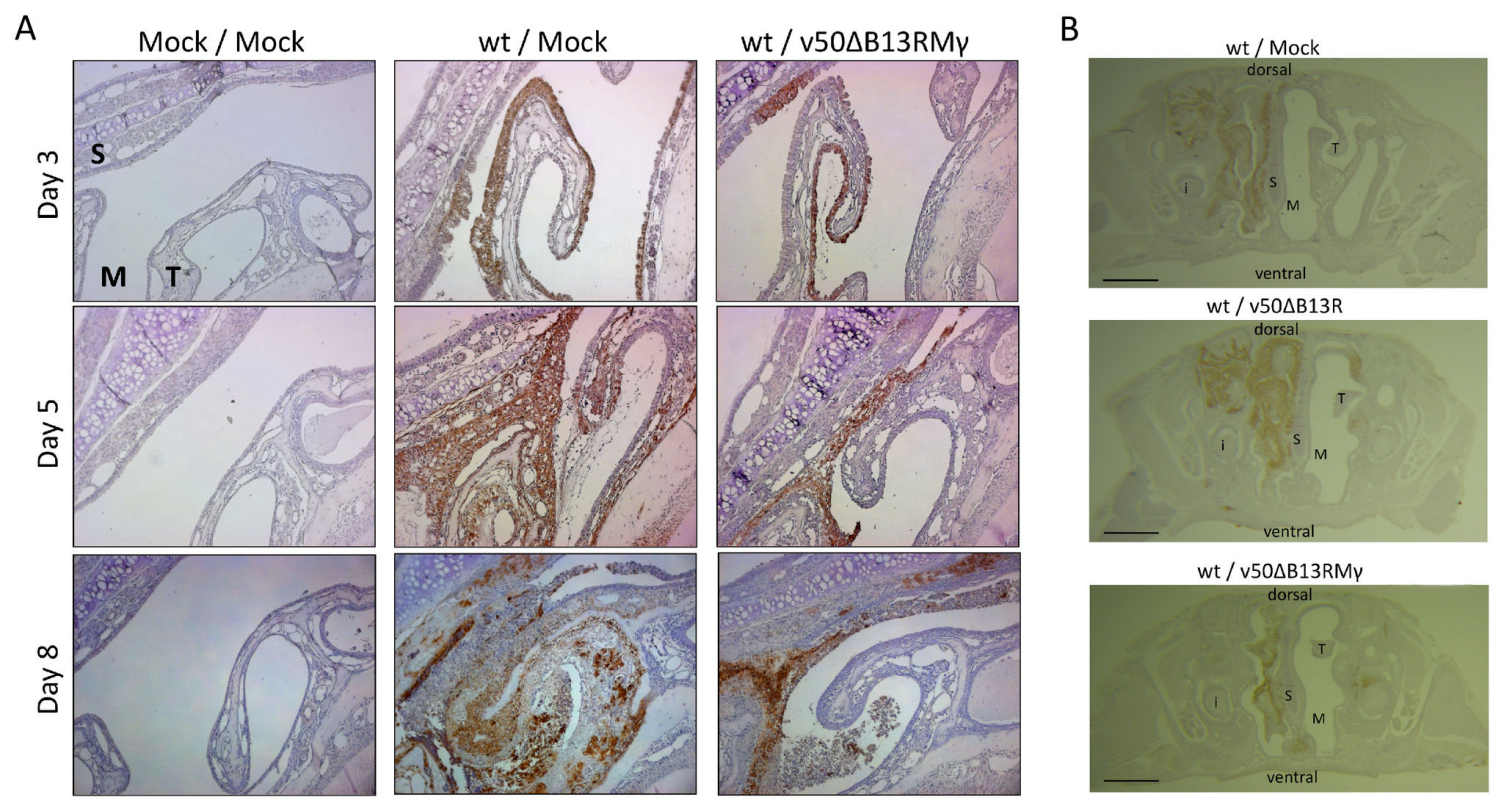
Histopathologic comparison and infection progression in the nasal cavity section. Mice were infected IN with 10^6^ pfu (~100 LD_50s_) of wt VACV and treated one day post-infection with 10^7^ pfu of v50ΔB13RMγ. Mice were sacrificed at 7-8 days post-infection. All representative sections were stained with polyclonal antibodies against VACV. S=septum, T=maxilloturbinate, M=meatus (air passage), i=incisor. (A) Maxilloturbinate section, 2 mm depth, 100X magnification (B) Whole section, 5-6 mm depth. 10X magnification. Lines indicate 200 μm length.

Histological analysis of nasal tissue was performed in order to determine differences in pathology following treatment with v50ΔB13RMγ. Staining for VACV antigen in a coronal section of the nasal cavity showed that in a wt VACV infection followed by mock treatment, virus replicated in the epithelial cell layer early during infection by day 3. As infection progressed, VACV replication moved into the underlying submucosal tissue and epithelial sloughing, tissue necrosis, and edema were evident (Figure 6A). By 8 dpi in mock-treated animals, the nasal cavity was unilaterally blocked and replication had progressed in both dorsal and lateral directions (Figure 6B). Additionally, in deeper sections (> 2mm depth) infection progressed in a posterior direction toward the olfactory bulbs (data not shown). In animals treated with v50ΔB13RMγ, VACV staining was observed at day 3 in the epithelial cell layer, however at days 5 and 8, a reduced progression of VACV staining into the submucosal tissue was observed as well as reduced levels of tissue necrosis and edema (Figure 6A). Photographs of the entire nasal passages show that in v50ΔB13RMγ-treated animals at 7 dpi VACV replication was evident along the airway epithelium but did not progress dorsally or laterally as compared to mock-treated animals (Figure 6B). In addition, in animals treated with the parental virus, v50ΔB13R, replication proceeded both dorsally and laterally in the tissue and stained more intensely, likely due to the extra virus added upon vaccination. Leukocyte influx into the nasal cavity was elevated in animals treated with v50ΔB13RMγ at 2 dpi (1.4 x 10^5^ cells) as compared to infected, mock-treated animals (3.2 x 10^4^ cells) and to mock-infected, mock-treated mice (6 x 10^3^ cells). However, numbers of leukocytes in the nasal cavity between the treated and untreated animals were similar by 4, 6 and 8 dpi (data not shown).

### Efficacy of therapeutic administration of v50ΔB13RMγ to animals infected with ECTV

**Figure 7:**
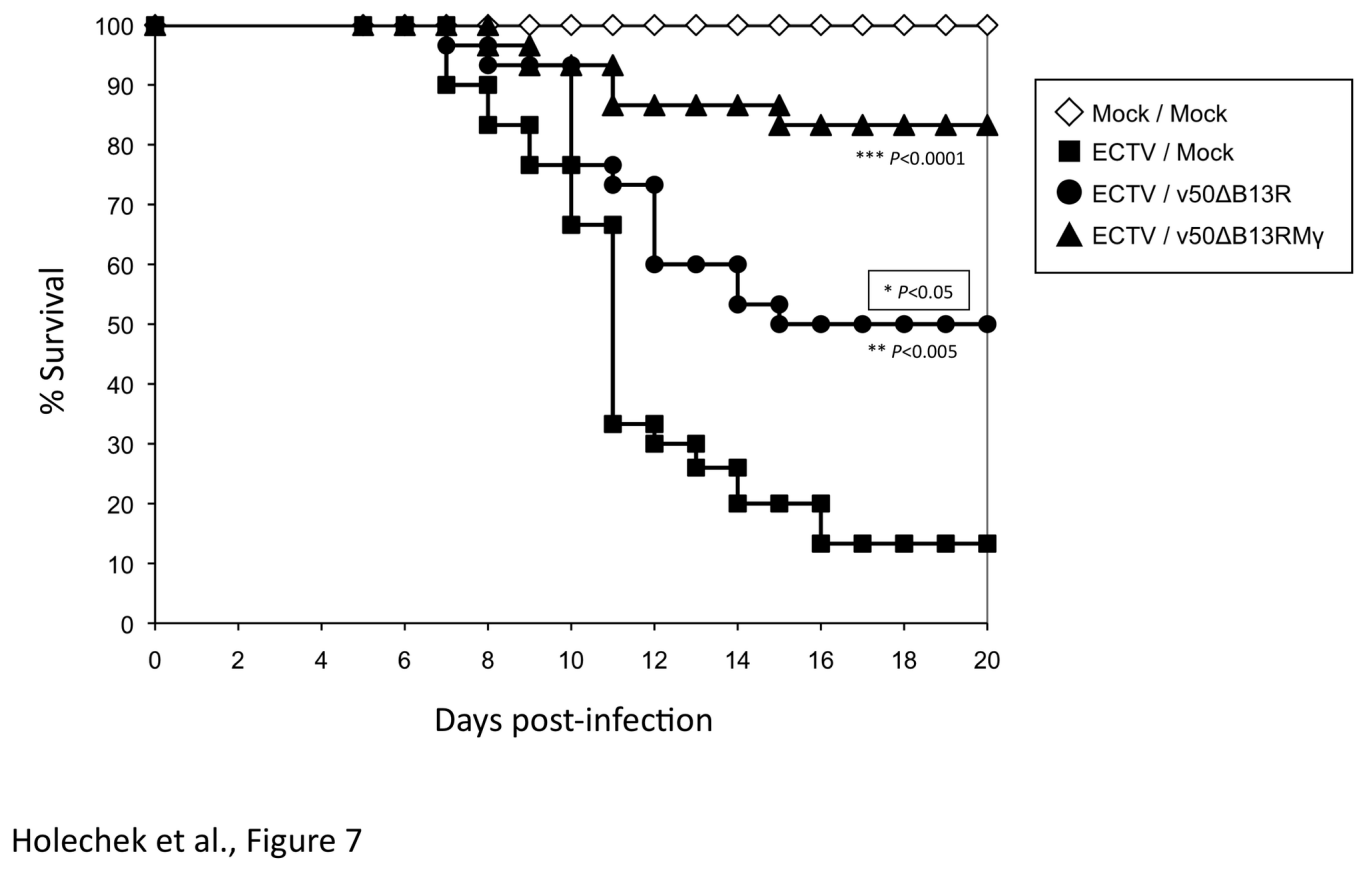
Protection of wt ECTV infected animals by post-exposure vaccination with v50ΔB13RMγ at one day post-infection. Groups of 30 C57BL/6 mice were infected intranasally with 5 x 10^3^ pfu (~50 LD_50s_) of wt ECTV. Animals were treated by FP route with saline (■), 3 x 10^7^ pfu of v50ΔB13RMγ (▲) or v50ΔB13R (●) at one day post-infection. One group of animals was mock infected and mock-treated (◊). The data were pooled from two independent experiments using group sizes of 10 and 20 mice. *P* values (log-rank test) show the significance of difference with respect to ECTV/Mock. The boxed *P* value shows the significance of the difference between v50ΔB13RMγ and v50ΔB13R.

ECTV infection which causes mousepox, has an LD_50_ of 90 pfu in C57BL/6 mice following IN infection, and models human smallpox in terms of the level of respiratory susceptibility [48]. In order to determine if v50ΔB13RMγ can protect animals against a lethal IN ECTV infection, C57BL/6 mice were infected IN with 5 x 10^3^ pfu of ECTV (55 LD_50s_) and treated by the foot pad (FP) route one day post-infection with 3 x 10^7^ pfu of v50ΔB13RMγ or v50ΔB13R. As shown in Figure 7, both v50ΔB13RMγ (*P*<0.0001, log-rank test) and v50ΔB13R (*P*<0.005, log-rank test) immunizations provided significant protection against a lethal ECTV IN infection with 83.3% and 50% survival, respectively, as compared to the mock-treated control group, however the protection afforded by immunization with v50ΔB13RMγ was significantly better than the control v50ΔB13R (*P*<0.05, log-rank test).

## Discussion

There are currently anecdotal studies regarding the efficacy of post-exposure vaccination protection of individuals when administered up to 4 days post-exposure [22]. The present work was intended to evaluate potential vaccine candidates for prophylaxis after exposure to a pathogenic orthopoxvirus. While other post-exposure prophylaxis studies have been done using replication-deficient and replication-competent VACV vaccine strains in order to protect against a lethal infection with VACV-WR or ECTV [23][24][49], our study is the first one that successfully uses a recombinant VACV expressing IFN-γ, v50ΔB13RMγ, for post-exposure protection. Our results suggest that IFN-γ is acting as a modulator of the immune response rather than an antiviral. 

Previous studies have demonstrated that VACV expressing IFN-γ can be used as an adjuvant as well as an attenuating agent for the production of live vaccines that are both safe and have high efficacy [50][51]. Moreover, expression of IFN-γ has also been used to influence the course of bacterial infection, where VACV as a vector was more effective than fowlpox [52]. Other viruses such as bovine herpesvirus-1 (BHV-1) have also been used to express IFN-γ and the stability of the BHV-1/IFN-γ virus as well as its immunomodulating effects studied upon primary infection and following reactivation of a latent infection [53]. Moreover expression of human IFN-γ by a simian immunodeficiency virus (SIV_HyIFN_) has shown reduced viral loads in the blood of rhesus macaque monkeys that were infected with this recombinant virus, even in the presence of a progressive deletion of the IFN-γ gene. These results suggest that the early immune system activation by SIV_HyIFN_ was able to confer protection against new emerging SIV viruses lacking full expression of the lymphokine [54]. Expression of IFN-γ in bacteria has also been studied. Administration of a recombinant IFN-γ produced in *E. coli* at the time of a primary immunization with vesicular stomatitis virus glycoprotein (VSV-G) for example, enhanced secondary antibody responses even at low doses [55]. The use of recombinant murine IFN-γ to prevent lethal respiratory VACV infection has also been successful [44] although the challenge dose of VACV was significantly lower than the ones used in this study.

IFN-γ is known to regulate the immune system by activating macrophages and neutrophils, enhancing NK cell activity, regulating B and T cell responses to antigens, stimulating specific cytotoxic T cells, promoting chemokine expression, as well as contributing to the protection against viral pathogenesis [26][44][45][46][47]. Mice bearing a disrupted IFN-γ gene have impaired production of macrophage antimicrobial products, reduced major histocompatibility complex (MHC) class II expression and are more vulnerable to infections against pathogens such as *Mycobacterium bovis* [56] and *Plasmodium falciparum* [57]. It has also been observed that during a VACV infection IFN-γ inhibits VACV replication through the induction of nitric oxide synthase (iNOS) in murine macrophages which leads to the production of nitric oxide (NO) from the guanidine nitrogen of L-arginine [58]. IFN-γ is the only known cytokine to induce NOS in macrophages, production of NO in turn affects DNA replication of VACV preventing virus particle formation [58][59]. As noted in this study, although all animals infected with wt VACV survived upon treatment with v50ΔB13RMγ, VACV was found present in all the organs examined suggesting that this virus is acting more as an immunoregulator than as an antiviral. Thus, v50ΔB13RMγ could be indeed orchestrating immune responses that could lead to less morbidity and mortality in this animal VACV model.

Several parameters were studied in order to optimize post-exposure protection in our animal model. IN infection was chosen for challenge as the respiratory route is considered to be the natural route of transmission for smallpox [60]. We found that the route of treatment was important, and IN treatment was the most effective in our VACV-WR model. Survival rate of animals infected IN with wt VACV and treated with v50ΔB13RMγ, especially into the same naris as the challenge, provided complete protection while the use of other routes of treatment reduced the survival rate to 40% (INON) and 20% (IM and SCA) (Figure 3). Similarly, in a study by Staib et al. (2006), mice infected IN with 5 x 10^4^ pfu (1 LD_50_) VACV-WR strain and then treated IM at 1, 2, 3 or 4 dpi with 10^8^ infectious units of the modified VACV Ankara (MVA) died by day 9 and had no effect compared with mock-vaccinated animals. Mice infected IN with 10^6^ pfu VACV-WR and treated by scarification with 10^6^ pfu VACV Elstree strain presented similar results [24]. The fact that in our model treatment is more effective when administered at the same site of the infection could be explained in part by the low replication levels of v50ΔB13RMγ in vivo (Figure 4C) [32]. This virus is attenuated and required a high dose (1 x 10^7^ pfu) for effective vaccination. We also found that the effectiveness of IN vaccination was dependent on expression of IFN-γ as treatment with the parental virus v50ΔB13R, only provided 20% efficacy in this model (data not shown). Moreover, although v50ΔB13RMγ does not spread well in tissues, it replicates to low levels in the nasal cavity of treated animals (Figure 4C), which could contribute, to an increase in the effectiveness of the prophylactic treatment. Additionally, the VACV-expressed IFN-γ binding protein (IFN-γ BP) has a low affinity for murine IFN-γ [61], making the availability of murine IFN-γ higher when used for treatment of a VACV-WR infection. Finally, the need to treat the same naris as the initial infection may, in a VACV model, show a need for direct immunomodulation of the inflammatory infiltrate which localizes at the primary site of infection. We have observed that infection of a single naris results in a unilateral region of viral replication and cellular infiltrate in the sinus cavity (Figure 5B). 

Treatment with a similar recombinant virus, v50ΔB13RMIL-18, which expresses IL-18 (an IFN-γ inducing factor), was partially successful in protecting mice from death and the viral spread to tissues was similar to wt VACV (data not shown). This partial protection could be due to the fact that VACV expresses an IL-18 binding protein, which binds IL-18 tightly [62][63]. Altogether these results suggest that post-exposure treatment in the VACV-WR model has more efficacy when administered in the same site of infection and that the expression of IFN-γ is more effective than treatment with conventional vaccine strains. 

Another parameter that was examined was time of post-exposure treatment. We demonstrate that post-exposure immunization with v50ΔB13RMγ is more effective in protecting mice against a lethal infection with VACV-WR when administered one day post-infection (Figure 2). Other studies have proven that post-exposure protection against a challenge dose of 1 to 3 LD_50s_ is possible when treatment with MVA is applied the same day of infection [23][24]. The action of IFN-γ by itself has been proven to be effective against a challenge dose of 8 LD_50s_ when treatment was administered one day before or the same day of infection. IFN-γ treatments started at 1, 2, and 3 dpi resulted in 90, 70, and 50% survival rates, respectively [44]. In contrast, our results show the efficacy of v50ΔB13RMγ for post-exposure vaccination prophylaxis following a challenge dose of 100 LD_50s_ of VACV-WR with 100, 40 and 20% survival when treatment was administered at 1, 2 or 3 dpi respectively (Figure 2). 

Despite seeing less morbidity and no mortality in the treated animals with v50ΔB13RMγ, we observed weight loss and viral replication in the organs that we examined. Weight loss has been correlated with fever and is a reliable method of determining relative pathogenesis [64]. Our study shows a continual weight loss in animals infected with wt VACV. However, animals treated with v50ΔB13RMγ lost a maximum of 25% weight and recovered rapidly (Figure 4) reaching a weight comparable to uninfected animals by day 18 (data not shown). While our data show that viral load and tissue distribution were similar for both untreated and treated groups up to 6 dpi, mice infected with wt VACV died by day 8 (Figures 4A and 4B). In our study, viral titers in most of the affected organs from treated animals diminished after day 6 and by day 10 wt VACV was found only in the nasal cavity, brain, and lungs (one animal with titers in all three tissues and one animal with titers in nasal cavity and lungs only) (Figure 4B). Viral replication of wt VACV in the nasal cavity, brain, lungs, ovary, spleen and liver has been previously observed [23][31][65]. Immunohistochemistry results in our VACV model showed less necrosis and edema blocking the air passage in all of the animals that were treated with v50ΔB13RMγ as compared to the mock-treated animals (Figure 5). These results correlate with a significantly lower viral load in the olfactory bulb of v50ΔB13RMγ treated animals (7.16 x 10^6^ pfu/g) at 6 dpi in comparison with mock-treated animals (1.53 x 10^8^ pfu/g) *P*=0.0062, one way ANOVA, data not shown). However all other tissues tested, including the nasal cavity, showed similar viral titers in treated versus untreated animals, and this suggests that the expression of IFN-γ is not acting as a direct antiviral. 

Upon treatment with v50ΔB13RMγ serum levels of IFN-γ were elevated in comparison to untreated or v50ΔB13R-treated animals. The higher levels of serum IFN-γ can be correlated with the low but measurable titers of v50ΔB13RMγ in the nasal cavity, brain, heart, and lungs of wt-infected mice and with the survival of these mice. A previous study using this virus has shown that infection with v50ΔB13RMγ alone results in undetectable viral titers as compared to v50ΔB13R, yet promotes potent antibody, T-helper and cytotoxic T cell responses [32]. It appears that treatment with v50ΔB13RMγ into the same naris following wt infection allows a low level of v50ΔB13RMγ replication and may at the same time promote a protective immune response. This has been observed during coinfection with MVA viruses expressing the HIV env gene and IFN-γ. In this study, the addition of MVA expressing IFN-γ upon infection similarly resulted in elevated serum levels of IFN-γ along with an increased CD8+ T cell response [66]. At the same time, MVA expressing IFN-γ did not affect the levels of viral gene expression in tissues, so the expression of IFN-γ appeared to be primarily immunomodulatory in effect.

Because IN treatment with v50ΔB13RMγ was shown to prevent death in mice infected with wt VACV, we performed an experiment in order to determine the efficacy of post-exposure protection in a heterologous mousepox model which has been useful for testing both antivirals and vaccines and mimics human smallpox due to its infection of the respiratory tract and blood viral load [67][68]. Post-exposure vaccination also mimics the human system since the vaccine would be given by the intradermal route. We challenged A/Ncr and C57BL/6 mice IN with a lethal dose of ECTV. Opposite to what we observed in the VACV-WR model, IN treatment with v50ΔB13RMγ was unable to prevent mortality in A/Ncr and C57BL/6 mice and all animals died by day 10 (data not shown). The footpad route was then chosen as the route of treatment as this mimics the method that would be used for vaccination in humans. In our study, survival was 83.3% when animals were treated 1 dpi with v50ΔB13RMγ, and we observed a 50% survival when vaccinating with the parental v50ΔB13R. Previous results have demonstrated that post-exposure protection against ECTV was dose and time dependent with similar protection of 83% at 1 dpi following ID vaccination with VACV Lister [23]. While VACV Lister proved to be effective in protecting animals from a lower dose challenge of ECTV (3-5 LD_50s_) in Paran's study, v50ΔB13RMγ was able to protect mice infected with a higher dose challenge of 55 LD_50s_. Both studies used ID prophylactic vaccination and the treatment with either VACV-Lister or the attenuated v50ΔB13R was sufficient to partially protect animals after IN challenge with ECTV. Although v50ΔB13RMγ has been shown to replicate poorly in tissues [32], its increased effectiveness upon prophylactic vaccination is likely due to a sufficient antigenic dose (3 x 10^7^ pfu) along with expression of IFN-γ. 

Differences between the VACV and the ECTV model may be due to the events following infection using different routes of inoculation as well as disease progression. Such differences have been previously reported in an ECTV infection model [23][69][70]. Moreover, failure to protect infected mice by IN treatment could be attributed to the fact that the IFN-γ BP expressed by ECTV has been shown to inhibit the biological activity of murine IFN-γ [71][72][73]. Thus, any IFN-γ synthesized in the nasal cavity would likely be inactivated by ECTV's IFN-γ BP. Efficacy of the foot pad treatment could be explained by v50ΔB13RMγ reaching a draining lymph node, expressing IFN-γ to enhance the adaptive immune response, which due to the extended incubation time of ECTV, had time to mount an effective immune response against wt VACV. It has been previously shown that strong antibody, CD4, and CD8 responses are generated upon infection by v50ΔB13RMγ in spite of the low replication levels *in vivo* [32]. While treatment with the parental virus v50ΔB13R had low efficacy in the VACV model, it had some effect in the ECTV model (Figure 6). This suggests either that the deletion of the serpin homolog B13R could be boosting the anti inflammatory response by allowing the proteolytic activity of caspase-1, potentially increasing IL-18 activity and increased IFN-γ expression, or simply that prophylactic vaccination with an adequate dose of VACV would allow a sufficient inflammatory response for rescue [35]. 

Overall our results suggest that v50ΔB13RMγ may be working as a stimulator and modulator of the immune response rather than as an antiviral due to the similar viral titers observed in tissues of untreated versus v50ΔB13RMγ-treated wt VACV infected mice. IFN-γ is known to mediate several immune responses including activation of macrophages and neutrophils, enhancement of the NK cell activity, regulation of B cell functions, stimulation of specific cytotoxic T cell immunity, chemokine gene expression, increase in expression of MHC class I and II proteins, leukocyte attraction to the site of infection as well as contributing to the growth, maturation and differentiation of many cell types [26][44][45][46][47]. While v50ΔB13RMγ is a highly attenuated virus, it is still able to induce humoral, T helper, and cell-mediated immune responses[32]. We speculate that following infection with VACV, v50ΔB13RMγ infects cells and initiates a rapid Th1 response, as indicated by the rapid influx of leukocytes at 2 dpi in treated mice compared to controls. This rapid response would have a "delay" effect on the infection giving the animal time to mount a more robust immune response against VACV. Further studies are necessary in order to determine the immunological mechanisms involved in this process and which cells are the key players. 

This work demonstrates that expression of murine IFN-γ by a recombinant VACV is able to confer a reduction in pathogenesis and prevent mortality in mice infected with a lethal dose of wt VACV as well as to reduce mortality in an ECTV model. Our results highlight the importance of IFN-γ as a modulator of the immune response for post-exposure prophylaxis. Immunization with a recombinant virus expressing IFN-γ is known to prevent viremia and death [44][46] as well as promote a potent immune response [32]. Thus, the use of v50ΔB13RMγ could be an effective way to optimize post-exposure prophylaxis against smallpox and other orthopoxviruses infections. Moreover, together with ST-246, CMX001, cidofovir and VIGIV, VACV expressing IFN-γ could be utilized as another tool for post-exposure prophylaxis treatment.
